# Massive iron overload and acute-on-chronic liver failure in a patient with Diamond–Blackfan anaemia: a case report

**DOI:** 10.1186/s12876-020-01468-9

**Published:** 2020-10-12

**Authors:** Guilherme Rossi Assis-Mendonça, Marlone Cunha-Silva, Mariana Franson Fernandes, Luiza Dias Torres, Monica Pinheiro de Almeida Verissimo, Marcelo Trevisan Neves Okano, Daniel Ferraz Mazo, Cristina Alba Lalli, Tiago Sevá-Pereira, Rafael Fantelli Stelini, Larissa Bastos Eloy da Costa

**Affiliations:** 1 Department of Pathology, University of Campinas (UNICAMP), Rua Tessália Vieira de Camargo, 126, Cidade Universitária Zeferino Vaz, Campinas, SP 13.083-887 Brazil; 2 Division of Gastroenterology (Gastrocentro), University of Campinas (UNICAMP), Campinas, Brazil; 3 Boldrini Children’s Center, Campinas, Brazil; 4 Department of Internal Medicine, Faculty of Medical Sciences, University of Campinas (UNICAMP), Campinas, Brazil

**Keywords:** Diamond–Blackfan anaemia, Haemochromatosis, Liver fibrosis, Acute-on-chronic liver failure, Heart failure, Autopsy

## Abstract

**Background:**

Genetic anaemias lead us to reflect on the classic ‘trolley dilemma’, when there are two choices but neither one is satisfactory. Either we do not treat anaemia and the patient suffers from chronic tiredness and fatigue, or we do treat it through blood transfusions, leading to iron overload, which is a quite harmful consequence.

**Case presentation:**

We present the case of a 34-year-old woman with Diamond–Blackfan anaemia (DBA). Bone marrow stem cell transplantation had not been accessible during her childhood, so she had been submitted to monthly blood transfusions throughout her life, leading to a hepatitis C virus infection (which was treated, achieving a sustained virological response when she was 18 years old), and secondary haemochromatosis. Despite chelation therapy, diffuse iron deposition was occurring in multiple organs, markedly in the heart and liver. Her serum ferritin was higher than 21,000 ng/mL and transferrin saturation reached 102%. When she faced heart decompensation, this congestive condition led to an acute liver injury overlapping pre-existing hepatic fibrosis. She progressed to haemodynamic and hepatic failure, with clinical features of acute-on-chronic liver failure (ACLF). Despite therapeutic optimisation, she died of respiratory insufficiency. An autopsy was performed and revealed the macroscopic and microscopic findings of a massive iron deposition in the liver, heart, lungs, spleen, bone marrow, thyroid and adrenal glands. We found marked advance of liver fibrosis (chronic damage), as well as necrosis of hepatocytes in zone 3 of the Rappaport acinus (acute damage), supporting the hypothesis of ACLF. The main feature responsible for acute liver decompensation seemed to be heart insufficiency.

**Conclusion:**

This is the first case reporting the sequence: DBA, multiple blood transfusions, secondary haemochromatosis, advanced liver fibrosis, heart failure, ACLF and death. A multidisciplinary team is essential to care for DBA patients, since there is a significant emotional burden related to the disease, which might impair an effective chelation therapy and lead to severe consequences due to iron deposition.

## Background

Diamond–Blackfan anaemia (DBA) is an autosomal dominant inherited disease characterised by pure erythroid aplasia and malformations, especially of the hands, face, heart and urogenital tract. The diagnosis is often made in the first year of life, during an investigation of reticulocytopenic anaemia with a paucity of erythroblasts in the bone marrow [1].

A long-term follow-up is needed due to the frequent presence of congenital anomalies. In addition, DBA patients have increased susceptibility for malignancies, such as leukaemias, carcinomas and sarcomas [1, 2]. The only definitive treatment so far is allogeneic bone marrow stem cell transplantation, ideally before the age of 10 years. However, some patients are not eligible and most of them are submitted to chronic glucocorticoid therapy or transfusion regimens [1]. Nevertheless, these therapies are not free of side effects. Glucocorticoids increase the rate of diabetes, cataracts and bone fractures, whereas chronic transfusions – even with iron chelation – may lead to secondary haemochromatosis, which is the leading cause of death in DBA [3].

We report the case of a patient with DBA who had been submitted to multiple blood transfusions since childhood. The consequence was massive iron deposition in the endocrine system, spleen, bone marrow, lungs, heart and liver. A fatal outcome was achieved in the context of an acute-on-chronic liver failure (ACLF).

## Case presentation

A 34-year-old woman was diagnosed with DBA when she was 4 months old. As bone marrow transplantation was not available during her childhood, she had been treated with corticosteroids and monthly blood transfusions, leading to a hepatitis C virus infection when she was 18 years old. At that time, a liver biopsy was performed and evidenced hepatic siderosis (grade 4), as well as mild fibrosis. She underwent hepatitis C treatment and reached a sustained virological response.

In addition, she had amenorrhoea with a further diagnosis of hypogonadotropic hypogonadism, hypothyroidism and diabetes mellitus. Her daily prescription included deferasirox, deferoxamine, insulin and levothyroxine, with irregular adherence to treatment. In April 2019, she presented a non-complicated tooth infection, which required treatment with amoxicillin. A few days later, she complained of fatigue, dyspnoea, jaundice and lower limbs oedema, which progressively worsened over the next 15 days, and she was referred to our centre on suspicion of acute liver failure.

Physical examination revealed bilateral jugular stasis, liver enlargement, ascites, jaundice and lower limbs oedema. Pulmonary sounds were abolished on the right base. There was no fever or signs of a tooth abscess.

Initial laboratory tests showed normocytic and normochromic anaemia (haemoglobin 9.4 g/dL, normal range [NR] > 12.0) with a reduced number of reticulocytes (20 × 10^6^/mm^3^, NR 50–100 × 10^6^) and thrombocytopenia (41,000/mm^3^, NR > 150,000). The levels of serum ferritin were very high (21,973 ng/mL, NR 13–150), with transferrin saturation of 102%. Liver enzymes were slightly elevated: aspartate aminotransferase 195 U/L (NR < 35), alanine aminotransferase 183 U/L (NR < 35), alkaline phosphatase 181 U/L (NR < 104) and gamma-glutamyl transpeptidase 84 U/L (NR < 40). Liver function tests were abnormal: total bilirubin 11.4 mg/dL (NR 0.3–1.2), direct bilirubin 5.6 (NR < 0.2), albumin 3.0 g/L, (NR 3.5–5.2) and international normalised ratio 1.81 (NR < 1.25). There was also a reduction in serum levels of sodium (132 mmol/L, NR 135–145), phosphate (2.9 mg/dL, NR 3.5–5.1), magnesium (0.99 mg/dL, NR 1.6–2.1) and parathyroid hormone (8.0 pg/mL, NR 15–65). Renal function, thyroid hormones, ceruloplasmin and gamma-globulin were normal. Viral serologies for hepatitis A and B were negative, as was the hepatitis C viral load.

**Fig. 1:**
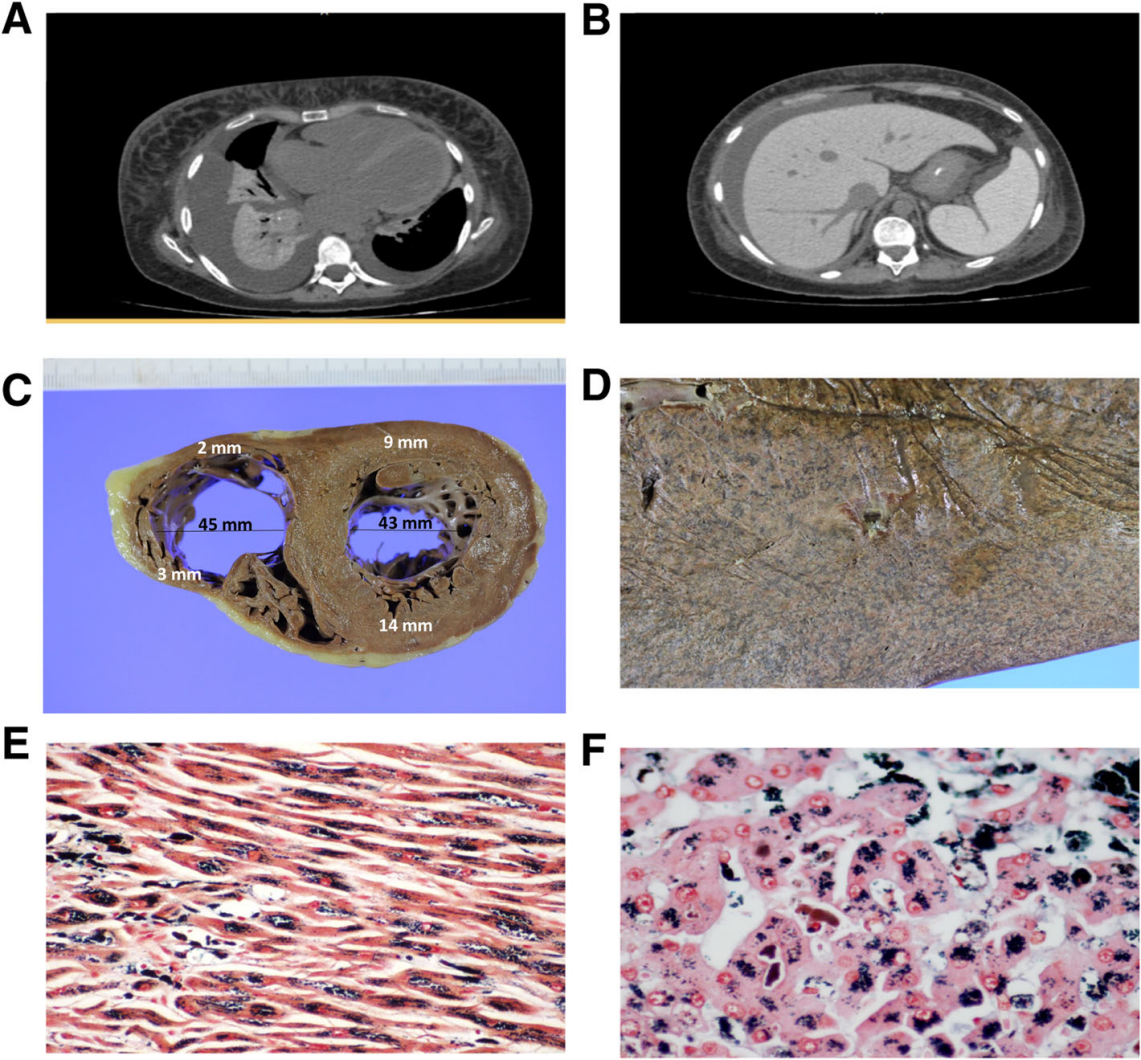
Radiological and pathological findings of the heart and liver. Axial sections of the computed tomography showing cardiomegaly and bilateral pleural effusions (**a**), as well as hepatomegaly and ascites (**b**). At post-mortem examination, the heart showed concentric left hypertrophy with dilation of the right ventricular cavity (**c**)**,** and the liver had a mottled appearance (“nutmeg liver”) (**d**). Massive iron deposition microscopically demonstrated in the heart (**e**) and liver (**f**) (Perls staining)

Abdominal ultrasonography showed hepatomegaly with a heterogeneous parenchyma, enlarged hepatic veins and ascites. The bile ducts and the portal vein were normal. Computed tomography showed heart enlargement, bilateral pleural effusions (Fig. 1a) without thrombosis in the pulmonary arteries, as well as hepatomegaly and ascites (Fig. 1b) without portal vein thrombosis. Further analysis of pleural effusion was compatible with transudate, according to Light’s criteria [4]. Ascitic fluid was not assessed because of the reduced volume. An echocardiogram revealed cardiomegaly, an ejection fraction of 28%, global dysfunction of the left and right ventricles, marked pulmonary arterial hypertension (medium 50 mmHg), mild tricuspid reflux and concentric left ventricular remodelling.

The patient presented sustained episodes of tachypnoea, desaturation and tachycardia. There was no response to treatment for decompensated congestive heart failure with diuretics and even the use of vasoactive drugs (dobutamine and noradrenaline). Blood, urine and pleural fluid cultures were negative. The patient received empirical antibiotics, despite no signs of active infection. Her serum bilirubin rose to 25 mg/dL. Although the liver morphology did not suggest advanced fibrosis by imaging exams, the patient manifested clinical features of ACLF. Unfortunately, her condition evolved to respiratory failure and she died on the 17th in-hospital day. An autopsy was then performed.

## Autopsy findings

On external examination, the patient’s body showed marked jaundice. After opening the body cavities, we measured considerable pleural effusions (1100 mL and 350 mL in the right and left hemithorax, respectively) and moderate ascites (2000 mL). Gross examination of the organs revealed concentric remodelling of the left ventricular wall, dilation of the right ventricle (Fig. 1c) and a mottled appearance of the liver, suggesting chronic passive congestion (Fig. 1d). Later, a microscopic examination revealed extensive iron deposits in the heart and liver, which was consistent with haemochromatosis (Fig. 1e–f). There was no mechanical obstruction of the biliary tree.

**Fig. 2:**
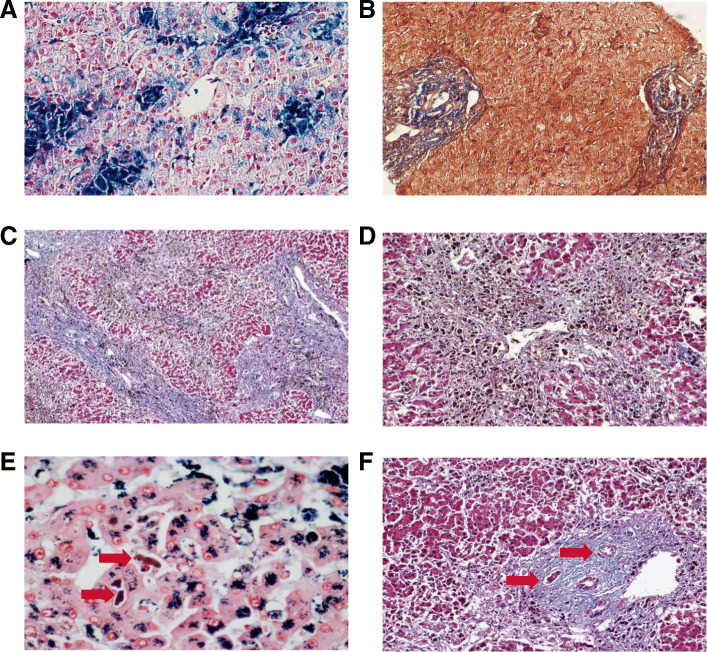
Hepatic Histological findings. The liver biopsy in 2003 already showed extensive iron deposits and a mild degree of fibrosis by Perls staining (**a**), and Masson’s Trichrome (**b**), respectively. A considerable increase in fibrotic septa was seen at the autopsy (**c**) (Masson’s Trichrome), as well as necrosis of hepatocytes near the centrolobullar vein (**d**) (Masson’s Trichrome). Bile plugs (arrows) were also seen (**e**) (Perls staining), in addition to ductular proliferations (arrows) in portal spaces (**f**) (Masson’s Trichrome)

We were able to access the patient’s previous liver biopsy (dated 2003), which already showed signs of chronic liver disease and iron overload (Fig. 2a–b). The autopsy findings revealed a marked advance of liver fibrosis (chronic liver damage), as well as necrosis of hepatocytes in zone 3 of the Rappaport acinus (acute damage) (Fig. 2c–d). Bile plugs – presumably from lysed hepatocytes – could be seen inside sinusoids, with ductular proliferations in zone 1 of the Rappaport acinus (Fig. 2e–f).

**Fig. 3:**
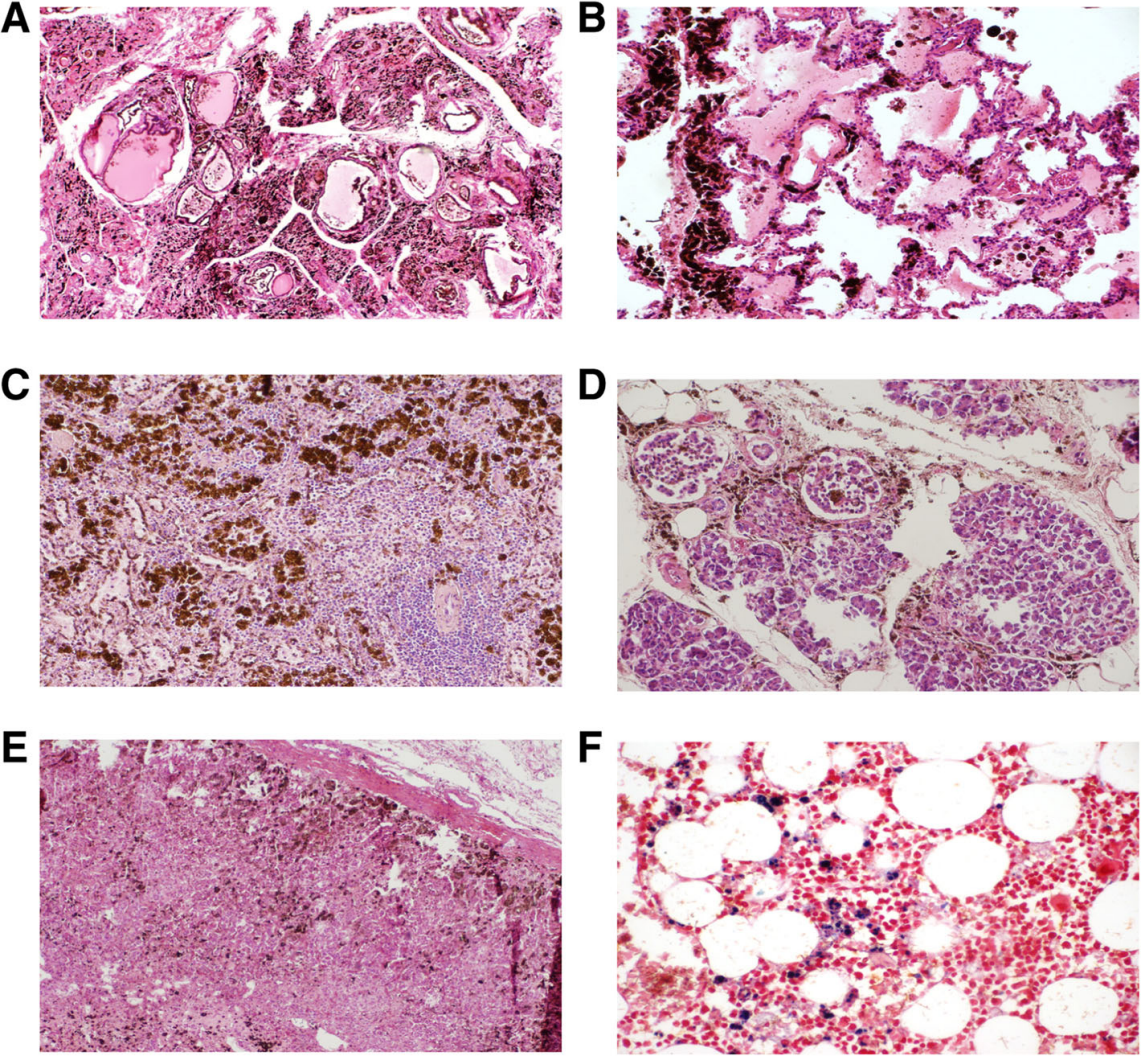
Representative images of haemochromatosis. Brownish spots compatible with iron deposits in the thyroid (**a**), lungs (**b**), spleen (**c**), pancreas (**d**) and adrenal glands (**e**) (Haematoxylin and eosin staining). Iron deposits in bone marrow macrophages (**f**) (Perls staining)

The spleen was enlarged at the autopsy (measurements of 17.9 × 10.5 × 4.0 cm and weight of 475 g), and microscopically presented an important congestion of the red pulp and haemosiderosis. Iron deposition was extended to the thyroid, lungs, pancreas and adrenal glands. Of note, deposits of this metal were present not only in macrophages but also in the parenchyma cells (Fig. 1e–f and Fig 3a–e).

The bone marrow was, as expected, hypocellular (around 50% of overall cellularity), with scattered erythroid precursors. No relevant alterations were seen in the granulocytic lineage, and mild cytological atypias were present in megakaryocytes. There was also an interstitial polytypic lymphoplasmocytosis. Finally, there were areas enriched on macrophages, which were also loaded with iron (Fig. 3f).

In the lungs, there was alveolar oedema in addition to the iron deposits. A final diagnosis of ACLF and congestive heart failure in a patient with multiorgan haemochromatosis could then be made, according to the patient’s clinical features and autopsy findings. The cause of death was set as respiratory failure secondary to pulmonary oedema.

## Discussion and conclusions

DBA is a rare, genetically driven anaemia, characterised by a high sensitivity of erythroid precursors to apoptosis [5]. Although there might be an initial response to corticosteroid therapy and eligibility of some cases for bone marrow transplantation, it is estimated that more than 30% of the patients will require chronic blood transfusions [6]. A leading complication of transfusion therapy is iron overload, an important cause of death among DBA patients [3]. This is the first case reporting an ACLF in a DBA patient in which acute liver damage (mainly related to congestive heart failure) overlapped chronic liver disease (promoted by iron deposits and consequent fibrosis).

The concept of ACLF encompasses the simultaneous acting of both acute and chronic insults to the liver, which can result from a wide range of triggers [7]. Recently, the need for including ‘cirrhosis’ in the definition of ACLF was discussed, since patients with advanced liver disease – but not necessarily cirrhosis – exhibit similar mortality rates compared to cirrhotic patients during the clinical course of ACLF [8]. Our patient, in spite of not being cirrhotic, already had evidence of chronic and progressive hepatic disease.

The difference between ACLF and decompensated cirrhosis is the systemic inflammatory response, which determines a slower recovery and a higher mortality in patients with ACLF compared to those adjudicated as having just decompensated cirrhosis [9, 10]. The precipitating factors may be the same [11]. Objectively, there is a score (CLIF-OF) based on six organ failures (hepatic, renal, haemodynamic, coagulation, neurological and respiratory) [12] to classify patients into the following categories: decompensated cirrhosis, grade 1, grade 2 or grade 3 ACLF. Mortality in 28 days in subjects with grade 1, 2 and 3 ACLF is about 20, 55 and 86%, respectively [9, 13]. Knowledge of the CLIF-OF score is quite important, as it is used to calculate the CLIF-C ACLF score, which is currently the best predictor of mortality in patients with advanced fibrosis and acute liver deterioration [14].

Our patient was admitted with an uncommon myriad of initial signs and symptoms that involved both the heart (dyspnoea, lower limbs oedema) and the liver (ascites, jaundice, coagulopathy). The autopsy was crucial for diagnostic confirmation, since there was a microscopic overlap of chronic and acute liver injuries. Clearly, most of her chronic heart and liver lesions were related to massive iron deposits. Furthermore, the main feature responsible for the acute liver decompensation seemed to be heart failure, as we found signs of the recent death of hepatocytes near the centrilobular vein (i.e. passive congestion). The role of cardiac failure as a precipitating event in ACLF has already been established; sadly, it is also a determinant of a worse prognosis, probably due to haemodynamic changes [15, 16].

Although potentially reversible, ACLF is a severe condition, and the outcome mainly depends on hepatic reserve and the prompt treatment of the dysfunctions. In our case, an advanced stage of liver fibrosis was observed at the autopsy, which compromised liver recovery and may have contributed to the outcome [7]. In addition, liver microscopy presented other features of the so-called ‘histological pattern 1’, with ductular proliferation, the presence of bile plugs and pericellular fibrosis, which can also determine unfavourable survival [7]. Finally, it is known that on ACLF, hepatocytes rely on haematopoietic stem cells to regenerate [7, 17]. This recruitment was probably impaired in the scenario of DBA and this may have been another contributing factor to a bad outcome.

There was a notable association between a tooth infection and the start of the major symptoms in our patient. Usually, a dental infection without systemic involvement is not expected to be a trigger for heart decompensation, but it has already been reported that oral infections are associated with the onset of pro-inflammatory states [18]. In patients with previous heart lesions, this inflammatory trigger may culminate in neurohormonal activation and acute events, such as thromboembolism or myocardial damage [19]. It is not clear whether this was really the trigger or if there was also another unidentified factor. It is noteworthy that in more than 30% of decompensated heart failures, no precipitating factor has been identified [20].

The management of patients with DBA is challenging. With the exception of cases eligible for bone marrow transplantation, there is no definitive cure [21]. Therefore, throughout the years of assisting these patients, clinicians often face moral decisions that illustrate the ‘trolley dilemma’ in medicine [22]. In this setting, chronic blood transfusions help, to some extent, in stabilising the patient’s anaemia. But even with chelation therapy, the inevitable consequence is a progressive iron deposition that may involve multiple systems [1]. Previously reported cases illustrate that secondary haemochromatosis is a constant feature in children and adults with DBA who are chronically submitted to blood transfusions, leading to dysfunctions in several organs and even to malignancies, such as hepatocellular carcinoma [23, 24].

The present case is quite representative, as we found iron deposits not only in the heart and liver but also in the lungs, spleen, thyroid, pancreas, adrenal glands and bone marrow. Therefore, the secondary haemochromatosis could explain the diabetes and hypothyroidism, and may have contributed to earlier respiratory failure. Interestingly, we found iron not only in macrophages, but also in parenchyma cells (e.g. cardiomyocytes, hepatocytes). This is a mark for long-term iron overload, and is a worrisome feature due to the toxic effect of this metal in parenchyma cells [23, 25].

To our knowledge, this is the first case reporting the sequence: DBA, multiple blood transfusions, secondary haemochromatosis, advanced liver fibrosis, heart failure, ACLF and death. A multidisciplinary team is essential to care for DBA patients, since there is a significant emotional burden related to a chronic disease, hard access to medications and arduous insertion in the labour market due to expected absences. This can lead to poor adherence to therapy and subsequent ineffective iron chelation, but the consequences of massive iron deposition may be severe. In fact, the retrospective study of Janov et al. (1996) [26] found a median overall survival of only 38 years for DBA patients, which reflects the difficulties in the management of these patients. In this context, we must emphasise that bone marrow stem cell transplantation should be performed in eligible patients as soon as possible. Less harmful treatments, such as gene therapy [27], are in development and may represent a new era in the management of DBA.

## Data Availability

Detailed information regarding this case is available from the corresponding author on reasonable request.

## Abbreviations

DBA: Diamond-blackfan anaemia
NR: Normal range
ACLF: Acute-on-chronic liver failure
CLIF-OF: Chronic liver failure (organ failure)
CLIF-C ACLF: Chronic liver failure consortium ACLF
